# *MYO5B* gene mutations may promote the occurrence of very early onset inflammatory bowel disease: a case report

**DOI:** 10.1186/s12920-024-01962-z

**Published:** 2024-07-16

**Authors:** Yue Lou, Yao Lv, Jindan Yu, Weizhong Gu, Ming Jiang, Jie Chen

**Affiliations:** 1 Gastroenterology Department, Children’s Hospital, Zhejiang University School of Medicine, National Clinical Research Center for Child Health, 3333 Bin Sheng Rd, Bin Jiang District, Hangzhou, Zhejiang 310052 China; 2 Pathology Department, Zhejiang University School of Medicine and National Clinical Research Center for Child Health, Hangzhou, Zhejiang China; 3 Institute of Genetics, Zhejiang University School of Medicine, Hangzhou, Zhejiang China; 4 Zhejiang Provincial Key Lab of Genetic and Developmental Disorder, Hangzhou, Zhejiang China

**Keywords:** *MYO5B*, Gene mutation, Very early onset inflammatory bowel disease

## Abstract

**Background:**

With recent advances in gene sequencing technology, more than 60 genetic mutations associated with very early onset inflammatory bowel disease (VEO-IBD) have been reported. Most of the genes are associated with immune deficiencies. The Myosin 5B (*MYO5B*) gene is primarily involved in cell motility and material transport which is associated with congenital intractable diarrhea and cholestasis. No studies have examined the relationship between the *MYO5B* gene and VEO-IBD. We report a case of a child with a mutation in the *MYO5B* gene who was diagnosed with VEO-IBD, then we investigated the association between the *MYO5B* gene and VEO-IBD.

**Case presentation:**

A 7-month-old baby girl with a chief complaint of “blood in the stool for more than 4 months and vaginal pus and blood discharge for 3 weeks” was diagnosed with VEO-IBD, and her symptoms improved after treatment with mesalazine. The whole-exome sequencing was performed with peripheral blood. Immunohistochemistry was performed on the terminal ileal tissue. Western blotting, quantitative polymerase chain reaction (Q-PCR) and immunofluorescence were performed with cultured organoid tissue from the terminal ileum. Whole-exome sequencing identified heterozygous missense of *MYO5B* variant of unknown significance (p. [I769N]; [T1546M]). Immunohistochemistry revealed a significant decrease in the expression of *MYO5B* protein in the terminal ileum of the child with *MYO5B* mutation; Q-PCR revealed a decrease in the mRNA levels of occludin and ZO-1 and both the mRNA levels and protein levels of *MYO5B* was downregulated in the patient. Immunofluorescence images showed that *MYO5B* gene mutation disrupted the apical delivery of transporters SGLT1, NHE3 and AQP7.

**Conclusions:**

MYO5B gene mutation leading to the downregulation of *MYO5B* protein may promote the occurrence of VEO-IBD by decreasing mRNA and protein levels of intestinal tight junction genes and dislocating the apical transporters.

**Supplementary Information:**

The online version contains supplementary material available at 10.1186/s12920-024-01962-z.

## Background

Inflammatory bowel disease (IBD) is a group of chronic gastrointestinal diseases of unknown etiology, of which children with IBD younger than 6 years of age are defined as VEO-IBD [1, 2]. 15% of children with IBD is VEO-IBD, and genetic sequencing techniques have identified single gene defects that commonly occur in children with VEO-IBD, including genes affecting intestinal epithelial barrier and response function, autoimmune function, excessive inflammatory response [3, 4].For example *IL-10*、*TTC7A*、*FOXP3*、*CYBA*, etc. [5].

*MYO5B* gene encodes a protein belonging to the type V myosin family, and is involved in cell motility and intracellular transport of various substances [6]. Deletion or inactivating mutation of *MYO5B* results in deficiency of apical sodium and water transporters leading to an imbalance in the Wnt/Notch signaling pathway, leading to altered enterocyte maturation and altered cell lineage differentiation [7]. *MYO5B* gene plays an important role in the intestinal epithelium, where it is involved in regulating the establishment of apical membrane circulation and apical delivery [8]. The sodium glucose co-transporter (SGLT1, SLC5A1) and sodium hydrogen exchanger isoform 3 (NHE3, SLC9A3) are expressed in the apical membrane of the small intestine and are participated in the exchange of water and Na^+ [9]^. The aquaporin water channels (AQPs) are transmembrane proteins specialized in the transport of water in the intestinal epithelium. *MYO5B* gene facilitates the delivery of transporters SGLT1, NHE3 and AQP7 to the apical membrane. Mutations in the *MYO5B* gene have been associated with microvilli inclusion body disease (MVID) and progressive familial intrahepatic cholestasis type 6 (PFIC6). MVID is a congenital autosomal recessive disorder that results in severe water- and electrolyte-transport-related diarrhea and dehydration in infancy, requiring parenteral nutrition and even intestinal transplantation. *MYO5B* gene deletion has been found to cause mislocalization of apical membrane proteins, resulting in water absorption dysfunction, diarrhea and microvilli atrophy, which then leads to the development of MVID [10].

However, whether *MYO5B* mutation could lead to the development of VEO-IBD remains unclear. We reported a case of a VEO-IBD child with a novel compound heterozygous mutation in *MYO5B* and uncovered the underlying cellular mechanism.

## Case presentation

**Fig. 1:**
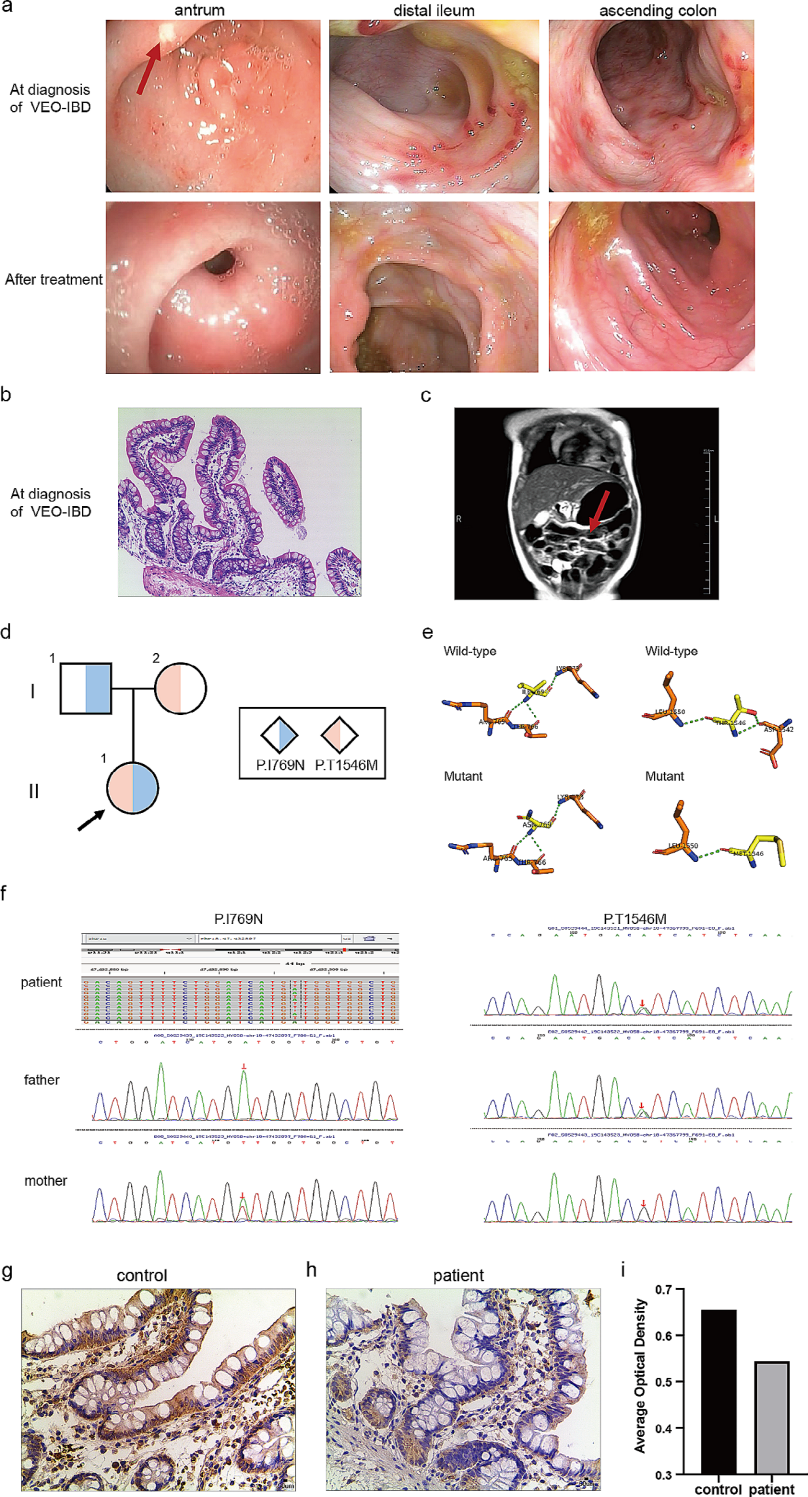
Identification of novel *MYO5B* mutation identified in children with VEO-IBD. (**a**) Gastroscopic and colonoscopic findings of the patient at the time of diagnosis of Crohn’s disease and after treatment; (**b**) Pathological findings of the patient at the time of diagnosis of Crohn’s disease, showing chronic inflammation; (**c**) MRE findings of the patient at the time of diagnosis of Crohn’s disease, with thickening of the intestinal wall of the left abdominal subintestinal canal and marked enhancement; (**d**) Pedigree chart of the patient; (**e**) Spatial structure changes of the patient and wild-type (WT) mutant loci; (**f**) Sanger sequencing results show mutations in *MYO5B* detected in the proband’s family; (**g**-**i**) Protein expression of *MYO5B* in the ileal terminus of this patient and the control child

A Chinese girl with a birth weight of 3.2 kg was delivered naturally at term. She was the first child of non-consanguineous healthy parents. At the age of 3 months, she had bloody stool, with fresh blood filament, accompanied with mucus, 3–4 times a day. After feeding with Neocate^®^ milk powder, the bloody stool of the child was relieved, however the blood filament was still visible. The child occurred a yellow-green vaginal discharge at the 7th month, which improved with antibiotic treatment, and three weeks later she developed with a purulent vaginal discharge with fecal-like flow visible through the vaginal. Her laboratory results showed increased white blood cells, increased lymphocytes, and increased C-reactive protein (CRP) (WBC 20.1*10^9/L, LY 66.1%, CRP 8.6 mg/L), without water-electrolyte disturbances. Irregular ulcers were seen under the esophagogastroscopy and ileocolonoscopy, and the surrounding mucosa was hyperemic and swollen (Fig. 1a). Pathological of intestinal tissue results showed highly normal terminal ileal villi with chronic mucosal inflammation (Fig. 1b). Magnetic resonance enterography (MRE) results showed that the intestinal wall of the left abdominal part of the child was thickened and the magnetic resonance signal was significantly enhanced (Fig. 1c). The perianal ultrasound showed a fistula in the anterior rectal wall 1.0 cm from the body surface, immediately behind the vaginal wall to the body surface. The patient was diagnosed as VEO-IBD and rectovaginal fistula and was subsequently treated with mesalazine. After 1 month, the therapy was added with prednisolone acetate because of recurrent pasty stools. 5 months later, the terminal ileal mucosa was congested and edematous, which was worse than before, so infliximab was used after that. One more month later, she received a second infliximab, which was immediately discontinued due to an allergic reaction and was maintained with a combination of mesalazine and thalidomide. After this treatment, the child achieved clinical remission and mucosal repair. The child’s stools returned to normal, and the gastroscopy and colonoscopy showed smooth mucosa (Fig. 1a).

At the age of 8 months, her peripheral blood was subjected to the whole-exome sequencing (WES) analysis and the Sanger sequencing technique was performed with her parents’ peripheral blood to detect mutations that might be associated with the disease. The sequencing results found two compound heterozygous mutations of unknown significance, P. I769N and P.T1546M, in *MYO5B*, which have not been reported so far. **(**Fig. 1d**)**. The AlphaFold 2 prediction software showed that the mutation of the isoleucine amino acid at position 769 to aspartic acid is a change from a non-polar amino acid to a polar amino acid. The mutation of threonine at position 1546 to methionine reduces the number of hydrogen bonds from three to one, decreasing stability and altering the three-dimensional structure of the protein. **(**Fig. 1e**)**. An additional figure file shows this in more detail [see Additional file 1]. P.I769N is originated from her mother and the isoleucine amino acid at position 769 was mutated to aspartic acid with a frequency of 0.0028374 in the normal population database **(**Fig. 1f**)**. P.T1546M came from her father and the threonine at position 1546 was mutated to methionine with a frequency of 0.0066192 in the normal population database **(**Fig. 1f**)**. We included a non-IBD child without *MYO5B* gene mutation by WES sequencing as s control. *MYO5B* protein expression level of the patient assessed by immunohistochemistry (IHC) staining in the biopsy tissue was lower than that of the control child **(**Fig. 1g-i**)**.

**Fig. 2:**
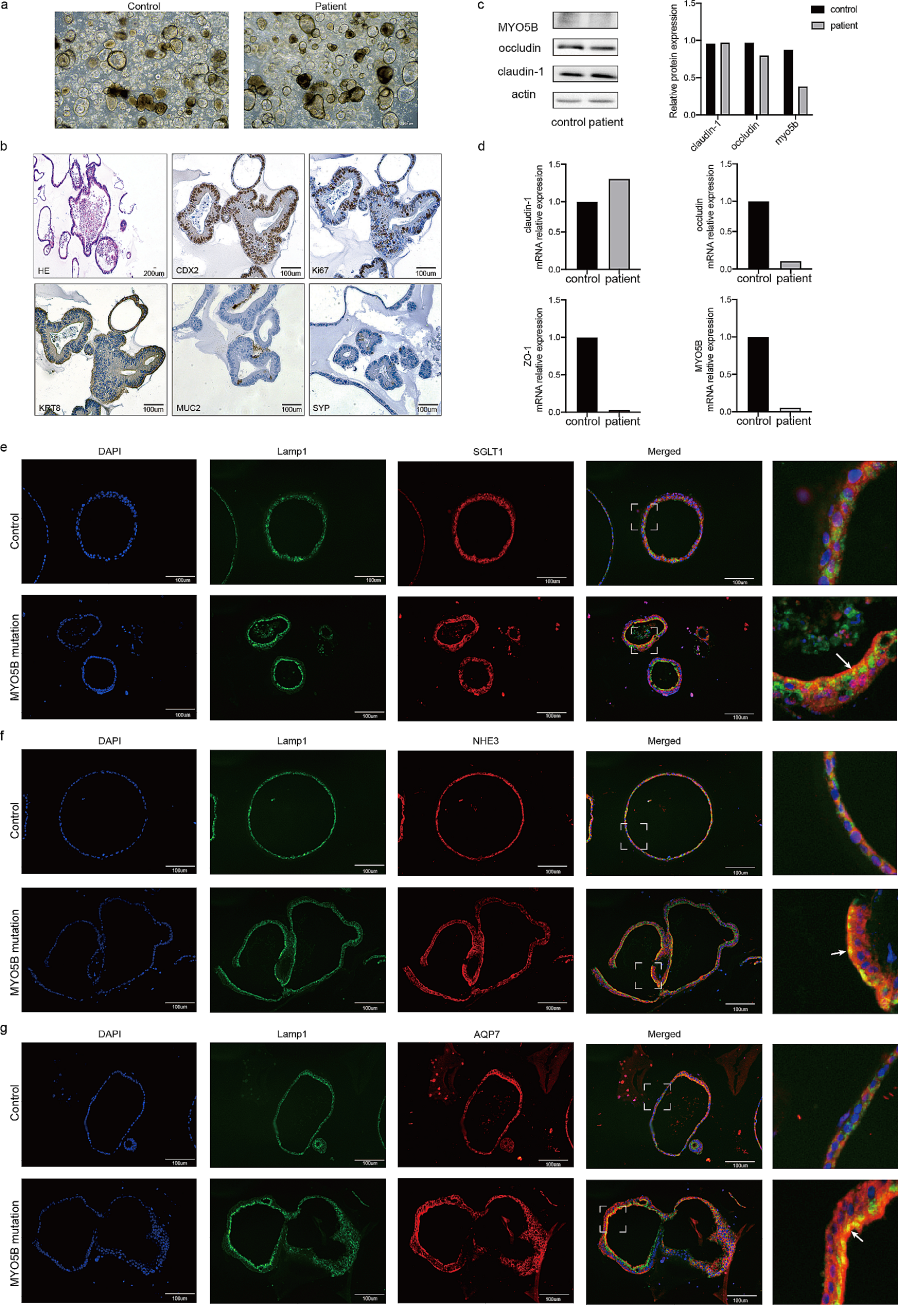
Mutations in the *MYO5B* gene cause changes in intestinal tight junction genes and apical transporters. (**a**) Organoids of this patient and the control child; (**b**) Cellular markers indicating successful organoids culture; (**c**) Expression levels of intestinal tight junction protein and *MYO5B* protein in this patient and the control (The blots were cut prior to hybridisation with antibodies, and groupings of the blot were cropped from different parts of the same blot); (**d**) mRNA expression levels of intestinal tight junction genes and *MYO5B* gene in this patient and the control; (**e**) Lamp1 lysosomes (green) in the control were beneath the brush borders labeled by SGLT1 (red). The *MYO5B* mutant patient showed a disorganized pattern of Lamp1 lysosomes, a decrease in SGLT1 in the apical membrane and an increase in SGLT1 in the cytoplasm. The yellow sites indicated by arrows indicated SGLT1-positive inclusions that co-localized with Lamp1 lysosomes; (**f**) In the *MYO5B* mutant patient, the expression of NHE3 in the apical membrane was reduced and the expression of NHE3 in the cytoplasm was greatly increased. The yellow areas indicated by arrows indicate NHE3-positive inclusions that co-localized with Lamp1 lysosomes; (**g**) In the control, AQP7 was located on the apical membrane above Lamp1 and was not expressed in the cytoplasm, while the *MYO5B* mutant patient showed abundant expression of AQP7 in the cytoplasm. The yellow areas indicated by arrows indicate AQP7-positive inclusions co-localized with Lamp1 lysosomes

To further evaluate the function, ileal derived organoids from the patient and the non-IBD control were established using small intestinal stem cells. We cleaned and minced the intestinal tissues for digestion and subjected the suspension to erythrocyte lysis, which was then coagulated and subsequently added to organoid medium and incubated in an incubator [11, 12]. Microscopic images showed mature organoids in culture at 2 weeks for both the patient and the control **(**Fig. 2a**)**. IHC imaging of caudal-related homeobox transcription factor 2 (CDX2) a specific cell marker, Ki67 a proliferation marker for the evaluation of cell proliferation, KRT8 a marker of columnar cells, mucin 2 (MUC2) production by goblet cells, synaptophysin (SYP) neuroendocrine markers in the patient’s biopsy were shown in Fig. 2B **(**Fig. 2b**)**. Western blotting results showed that compared with the control, the expression of *MYO5B* protein was down-regulated, the expression of intestinal tight junction protein occludin was decreased, and claudin-1 was not significantly changed. **(**Fig. 2c**)**; PCR results showed that the mRNA levels of occludin, ZO-1 and *MYO5B* in the patient were decreased compared with the control, and claudin-1 was not significantly altered **(**Fig. 2d**).** An additional figure file shows this in more detail [see Additional file 2]. Immunofluorescence imaging of organoids revealed that compared with the control, the disorganized pattern of the lysosomal marker Lamp 1 was observed in the patient, and the co-localization of the apical transporter SGLT1 with Lamp 1 was increased, which indicated that SGLT1 was abundant in the cytoplasm and reduced in the apical membrane **(**Fig. 2e**)**. NHE3 and AQP7 also showed similar results **(**Fig. 2f-g**)**.

## Discussion and conclusions

We identified a novel, double allelic mutation in the *MYO5B* gene in a child with VEO-IBD. This mutation may have an impact on the pathogenesis of VEO-IBD. The *MYO5B* gene is located on chromosome 18q21.1 and has 40 exons, encoding *MYO5B* containing 1848 amino acids. Rab proteins are the largest family of membrane traffic used to form multiprotein complexes on different vesicular membranes to regulate specific membrane trafficking pathways, and *MYO5B* is an effector of Rab8a, Rab10 and Rab11a [8, 13]. Rab proteins in different combinations with *MYO5B* control different membrane trafficking pathways. It has been reported that the *MYO5B* gene mutation cause downregulation of the *MYO5B* protein and dislocation of the apical transporters, leading to the development of MVID [9, 14]. A subset of patients with *MYO5B* deficiency develop cholestasis [15, 16]. Whether mutations in *MYO5B* are associated with the development of VEO-IBD is unclear and requires more in-depth mechanistic investigation. In this case, the patient combined with *MYO5B* gene mutations had a localized intestinal ulcer on colonoscopy. The ulcer was not deep and the surrounding mucosa was erythematous, and the patient could maintain long-term clinical remission and receive endoscopic mucosal improvement after the combination therapy of mesalazine and thalidomide, which supported the diagnosis of VEO-IBD. In particular, the protein and mRNA levels of *MYO5B* were downregulated and the apical transporters SGLT1, NHE3, AQP7 was misplaced in this patient, who did not show water-electrolyte imbalance or villi atrophy, which suggested that the *MYO5B* gene mutation did not cause MVID in this patient. To investigate whether mutations in the *MYO5B* gene disrupt intestinal mucosal barrier function, we examined the expression of several tight junction (TJ) mRNAs and protein levels. The intestinal mucosal barrier is mainly supported by epithelial cells interconnecting with each other, from apical to basement membranes in the order of TJ, adherens junction, bridges, and gap junctions. TJ consists of tight junction proteins, which play key roles in maintaining the function of intestinal epithelial barrier, and these proteins are Claudin, occludin, zonula occludens (ZO) and junction-associated molecules, among others. From our results, the mRNA levels of occludin and ZO-1 were decreased, which indicated that the intestinal barrier function was impaired in the child. The decreased protein and mRNA levels of *MYO5B* gene in this child indicated that these two mutation sites (c.2306T > A(P.1769 N), c.4637 C > T(P.T1546M)) caused the decreased expression of *MYO5B*. Furtherly, mutations of *MYO5B* gene led to a large expression of apical transporters *SGLT1*, *NHE3*, *AQP7* in the cytoplasm and blocked the transport traffic, resulting in a decrease in the expression on the apical membrane. It has been found that when the intestinal barrier function is disrupted, the expression of SGLT1 and NHE3 decreases, which affects Na^+^ absorption and leads to a disturbed metabolic state, while the exact relationship between the two is yet to be further investigated (17–18). The incidence of this heterozygous mutation in *MYO5B* is about 0.001%, and there are no more studies reporting that *MYO5B* heterozygous mutations leading to the development of VEO-IBD, which needs to be verified by more clinical cases.

This study aimes to investigate the relationship between the *MYO5B* gene and VEO-IBD. Overall, the findings showed that mutations in the *MYO5B* gene reduce the expression of *MYO5B* protein and mRNA, affect the transport of apical transporters, and reduce mRNA and protein expression of the intestinal tight junction genes. Therefore, we speculate that *MYO5B* gene leading to the downregulation of *MYO5B* protein may promote the development of VEO-IBD by causing mislocalization of SGLT1, AQP7, NHE3 and decreasing intestinal tight junction protein expression to disrupt water-sodium exchange and intestinal barrier function in the intestinal epithelium. Impaired intestinal barrier function is an important feature of IBD [19]. *MYO5B* gene mutation will cause the disruption of intestinal tight junction, and the specific mechanism through which it plays a role needs further exploration.

An additional text file shows more details [see Additional file 3].

### Electronic supplementary material

Below is the link to the electronic supplementary material.

Supplementary Material 1

Supplementary Material 2

Supplementary Material 3

Supplementary Material 4

## Data Availability

The datasets analysed during the current study are available in the [ClinVar] repository, [SCV005038745].

## Abbreviations

MYO5B: Myosin 5B
VEO: IBD-Very early-onset inflammatory bowel disease
Q: PCR-Quantitative polymerase chain reaction
MVID: Microvilli inclusion body disease
IHC: Immunohistochemistry
WB: Western blotting
IF: Immunofluorescence
CRP: C-reactive protein
WBC: White blood cells
WES: Whole-exome sequencing
SGLT1: Sodium glucose co-transporter
NHE3: Sodium hydrogen exchanger isoform 3
AQPs: Aquaporin water channels
MRE: Magnetic resonance enterography
CDX2: Caudal-related homeobox transcription factor 2
MUC2: Mucin 2
SYP: Synaptophysin
TJ: Tight junction
ZO: Zonula occludens
