# Supplementary figures and images for: MYO5B gene mutations may promote the occurrence of very early onset inflammatory bowel disease: a case report
Source: BMC Med Genomics. 2024 Jul 16;17:187. doi: 10.1186/s12920-024-01962-z (PMC11250955; doi:10.1186/s12920-024-01962-z)

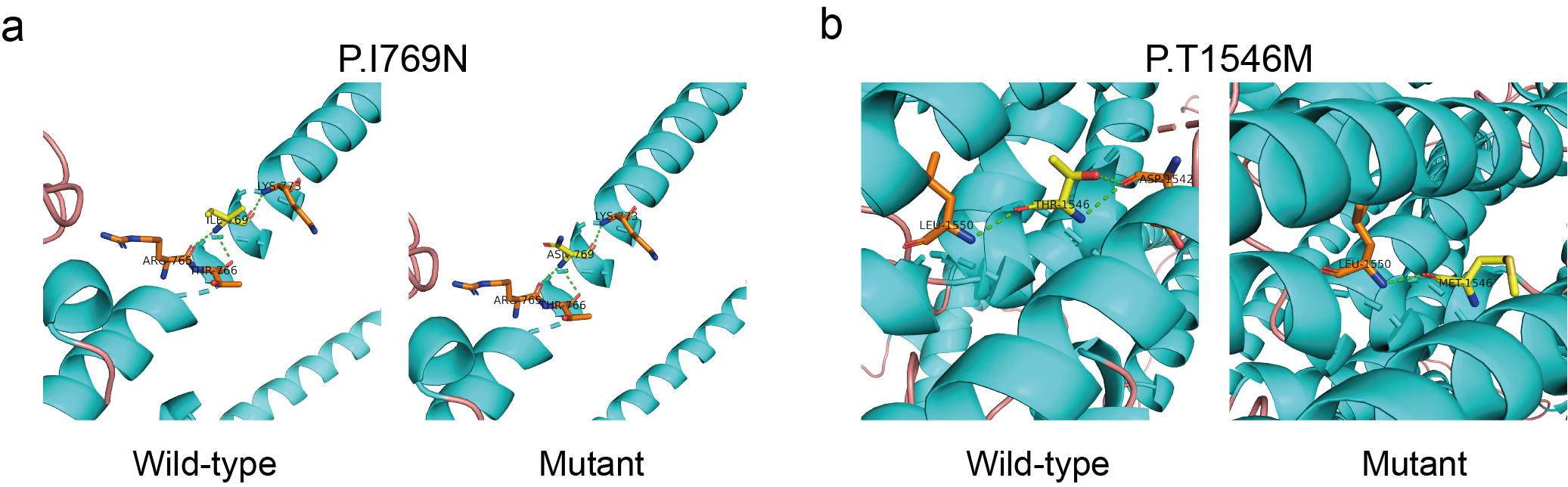

Supplement: Supplementary file 1 — Supplementary Material 1 [file 12920_2024_1962_MOESM1_ESM.png]

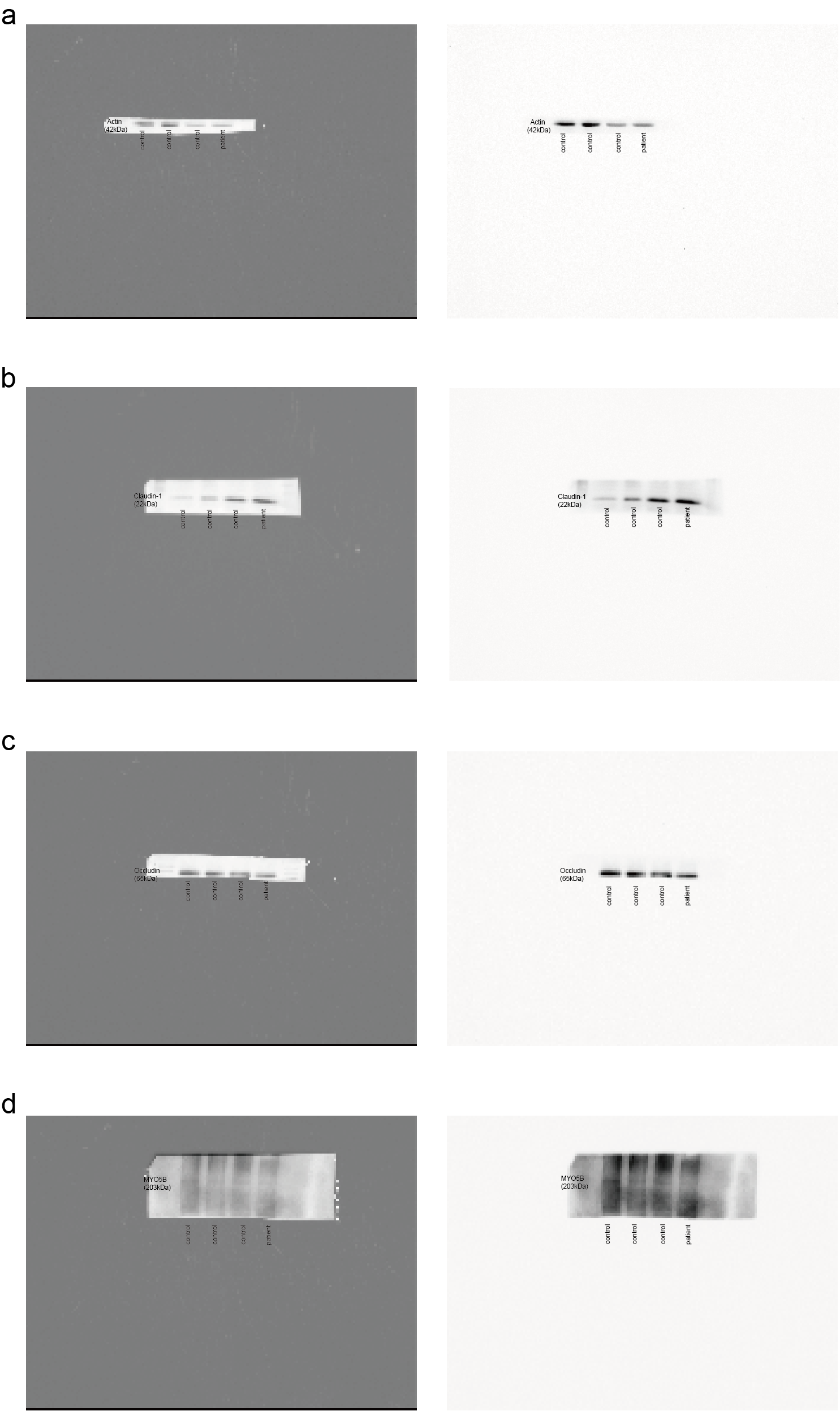

Supplement: Supplementary file 3 — Supplementary Material 3 [file 12920_2024_1962_MOESM3_ESM.png]
