# Supplementary material for: MYO5B gene mutations may promote the occurrence of very early onset inflammatory bowel disease: a case report
Source: BMC Med Genomics. 2024 Jul 16;17:187. doi: 10.1186/s12920-024-01962-z (PMC11250955; doi:10.1186/s12920-024-01962-z)
Supplement: Supplementary file 2 — Supplementary Material 2 [file 12920_2024_1962_MOESM2_ESM.docx]

**Methods**

**1.Study aim, design and setting of the study**

The study aimed to explore the relationship between MYO5B gene and VEO-IBD. The study took place at the children’s hospital of Zhejiang university school.

**2.Characteristics of participitans**

We analyzed the clinical manifestations, MRI results, gastroenteroscopy, and pathological results of the children with MYO5B gene mutation, and conducted relevant experiments on the intestinal mucosa.

**3.Immunohistochemistry staining**

IHC staining was performed according to the DAKO EnVisionTM kit instructions. After dewaxing, the samples were incubated with methanol containing 30% H2O2 for 20 min to block endogenous peroxidase activity, then immersed in 0.01 mol/L citric acid buffer (pH 6.0), microwave heated at 100℃ for 20 min, rinsed with distilled water three times, and sealed with 1% bovine serum albumin for 30 min. Overnight at 4℃, MYO5B Antibody (ab190096, Abcam) was diluted at 1:250. Then the second antibody (Dakocytomation Company, Denmark) was used at 37°C for 30 minutes. The specimen was rinsed 3 times with phosphate buffered brine (PBS) followed by color display with DAB (Dakocytomation Company, Denmark) for about 5 min.

**4.Organoid culture**

Tissue was minced and digested. The pellet was resuspended in Matrigel/BME (estimate 10ul pellet solution in 70 ul Matrigel/BME). 40 ul drops of BME-cell suspension were allowed to solidify on prewarmed 24-well suspension culture plates at 37℃ for 30 min. Add 400 uL of organoid medium and culture in humidified 37℃ / 5% CO2 incubators. Medium was changed every 3-4 days and organoids were passaged every 1-4 weeks. Organoids were mechanical dissociated by 10ul pipette when resuspened in 2 mL TrypLE Express, incubation for 1-5 min at room temperature. Neutralizing by DMEM/10% FBS and centrifugation at 400 rcf . Organoid fragments were resuspended in cold BME and reseeded as above at ratios (1:1 to 1:6) allowing the formation of new organoids. Cryopreservative medium (serum free) (CELLBANKERTM 2, ZENOAQ, 170905) was used for organoids cryopreservation.

**5.Western blot analysis**

Organoid tissues were weighed and homogenized in RIPA extraction buffer (Solarbio, China). The homogenate was centrifuged at 4°C for 15 min at 15,000 g, and the supernatant was collected. Protein concentration was quantified using the BCA Protein Assay Kit (Solarbio, China) according to the manufacturer's instructions. Proteins were separated with 6%+12% SDS polyacrylamide gels and then transferred to PVDF membranes. The membranes were blocked with 5% skim milk for 1 hour and then immunoblotted with primary antibodies against claudin-1 (RT-1141, Huabio), occuludin (#68534, CST), and MYO5B (ER60872, Huabio) at 4°C overnight. The membranes were then incubated with the secondary antibodies for 1 h at room temperature and the bands were observed using the ECL kit (P0018A, Beyotime). β-actin was used as a reference gene.

**6.Quantitative polymerase chain reaction**

RNA was extracted from the organoid tissues by using TRIzol reagent (Takara, Japan). Total RNA was reverse transcribed using HiScript III Reverse Transcriptase (Vazyme, China). qRT-PCR was per- formed using SYBR Premix Ex Taq in the StepOne Plus^TM^ Real-Time PCR System (Applied Biosystems) using cDNA. The relative mRNA expression was calculated using the comparative cycle method (2−ΔΔCt). GAPDH served as internal reference genes.

Claudin-1:

Forward: 5ʹ- TCTGGCTATTTTAGTTGCCACAG-3ʹ,

Reverse: 5ʹ- AGAGAGCCTGACCAAATTCGT-3ʹ;

Occludin:

Forward: 5ʹ- GACTTCAGGCAGCCTCGTTAC-3ʹ,

Reverse: 5ʹ- GCCAGTTGTGTAGTCTGTCTCA-3ʹ;

ZO-1:

Forward: 5ʹ- ACCAGTAAGTCGTCCTGATCC-3ʹ,

Reverse: 5ʹ- TCGGCCAAATCTTCTCACTCC-3ʹ;

MYO5B:

Forward: 5ʹ- AGTCCATCATAGTCAGTGGGG-3ʹ,

Reverse: 5ʹ- CCAACGGTGGCGAAATAGC-3ʹ;

**7.Immunofluorescence**

Sections of paraffin- embedded tissue were stained by Lamp1 antibody (#15665, CST), SGLT1 antibody (bs-1128R, Bioss), NHE3 antibody (bs-8601R, Bioss), AQP7 antibody (bs-2506R, Bioss) and DAPI (solarbio, China).
