# Supplementary material for: MYO5B gene mutations may promote the occurrence of very early onset inflammatory bowel disease: a case report
Source: BMC Med Genomics. 2024 Jul 16;17:187. doi: 10.1186/s12920-024-01962-z (PMC11250955; doi:10.1186/s12920-024-01962-z)
Supplement: Supplementary file 4 — Supplementary Material 4 [file 12920_2024_1962_MOESM4_ESM.docx]

**Additional file 1.png**

Fig s1. Predicted three-dimensional structure of the protein with mutations in the *MYO5B* gene

(a)*MYO5B* (P.I769N); (b) *MYO5B* (P.T1546M)

**Additional file 2.png**

Fig s2. The original unprocessed blot images of the patient and controls

1. Actin; (b) Claudin-1; (c) Occludin; (d)*MYO5B*

(Groupings of the blot were cropped from different parts of the same blot. Since the blot was cut prior to hybridisation with antibodies, we apologize for not being able to provide a complete uncropped image of the blot.)

**Additional file 3.docx**

Specific experimental methods
